# Anodyne Remedies

**Published:** 1903-07-15

**Authors:** N. S. Hoff


### ANODYNE REMEDIES.

BY N. S. HOFF, D.D.S.

Read before the Central Michigan Dental Association, May 14, 1903.

The meaning of the term Anodyne is "to be without pain." Therapeutically, it is that which assuages or relieves pain. To define the term for our purpose, we must consider the therapeutic application of this class of remedies as used in dental practice. They are sometimes referred to as Analgesic or Antalgic Remedies.

Anodynes may properly embrace all agents which will relieve pain in any measure, or by any method. Somnifacients, Anesthetics, Narcotics, Obtundents, Demulcents, Emollients, Sedatives, Correctives of various kinds are examples, because they may be employed to overcome painful conditions. A narcotic may be used as an anodyne remedy, but it may also be used as an anesthetic. An anesthetic is however rarely used to overcome pain, but to prevent it. For convenience we may separate the Anodyne Remedies into two classes: Those having a local use only and those having general or systemic application. We shall however find that some anodyne remedies have both a local and systemic action.

Local Anodynes are those which act because of physical or mechanical properties—Heat, Cold, Electricity.

They may be remedies of the chemical form; used easily as local applications for local effects, or to produce local effects from central or systemic action. We also have operative procedures, including all surgical means used to stop pain.

Systemic Anodynes.—The systemic anodynes are those which are used in the drug form having properties which overcome pain from a central action upon the nervous system through organic functions. The anesthetics might form one class; the narcotics another class; the cardiants, or those remedies which affect the blood circulation, a third class; and a fourth class the organic stimulants which are used to produce revulsive effects by depleting tissues which are locally congested, through some other organ or tissue which the drug stimulates inordinately or at least to the extent that it eliminates plethoric conditions which cause pressure and pain.

It is only when some extraordinary impression is made on the nervous system or the organs influenced by it that it becomes irritable or painful. The nerves of sensation of the mouth and face largely pass directly into the skull to the brain without passing through the spinal cord—as do the nerves of the trunk—and dental pains are generally severe and without systemic complications. Curing toothache by stopping the cavity, or treating the tooth only, is but one method. Pain may be felt in exposed dentine, but not be due to it, but to an impediment on the nerve trunk at some point in its course. Intense toothache is not always due to exposure of the dentine, but possibly to an internal nervous disorder. This is particularly true of neurasthenic people; and treatment of the tooth itself in such a case, with the idea of allaying the pain, would be futile. We must comprehend pathologic conditions and be able to determine the seat of the pain before we can apply intelligent treatment. If we know there is pressure on the nerve tract due to a growth in the bones through which the nerve passes, we may operate for its cure. If the pain is in the brain, we may anesthetize the patient and secure temporary relief; or we can narcotize with a general nervous sedative and overcome pain by relieving the irritability in the brain. For pain due to irritation of the peridental membrane we can inject into the membrane a little cocaine and temporarily relieve the pain, but this may not cure the trouble. We can also give a general anesthetic and anesthetize the brain so that our patient feels no pain. In other words, the exciting cause of pain, which may be anywhere in the nervous tract, must be reached to get permanent results. We apply these remedies to remove permanently the source of irritation if possible, but it is not always possible to do this, and as an expedient have to temporarily deaden the perceptive senses.

The first effort should be directed toward removing the local cause if possible, when the pain is of peripheral origin, thus preventing its effect upon the cerebrum, and other possible systemic effects. This we might do by cutting the nerve which carries the pain stimulus to the brain, but is not often practicable since it destroys the physiological function of the nerve, and also because it is difficult to locate and isolate in the facial tissues the particular nerve involved; and it would necessarily involve other important nerves. It is however practiced in the extirpation of live pulps. Nerves are sometimes stretched, but this is also an extreme procedure. A much preferable procedure is to narcotize without shock either the nerve endings in the inflamed region of the cerebrum, or the nerve tract. Morphine has the property of narcotizing the nerve ends, its trunk and the perceptive centers in the brain, but has most influence on the brain centers. Atropine excites the brain centers while it narcotizes the nerve trunk, and particularly the nerve endings; when given internally it produces a depression of feeling at the periphery while the brain is excited. Because it produces this internal stimulation, atrophine is usually classed as a systemic excitant. Cocaine has largely the same centric action as atropine, but has a most powerful peripheral and local narcotizing action. Probably this is the most profound local narcotic we have, but has such stimulating effects on the brain that reflex impressions from other parts of the body will be greatly magnified, while the cocainized area will be obtunded. Cocaine is a systemic poison only when it produces centric paralysis. Cocaine has experienced a good deal of disrepute because careless operators have ignored these facts in using it; they have failed to use it in such a way as to produce the greatest amount of local paralysis with the least amount of central excitement. To do this cocaine must be used in sufficient quantity to completely paralyze the reflexes in the tissue upon which the operation is to be made. If only partly paralyzed a reflex shock results from the already excited brain centers, and sensations of hysteria with other disagreeable nervous and organic symptoms ensue. In such cases the blame is the operators'. Small doses, but sufficient, should be used and in the best way to secure the greatest amount of local paralysis. The disagreeable phenomena come from not recognizing the effects of the drug on the central system.

Chloral and Gelsemium seem to have especial influence on the fifth nerve; they are more used than any other remedies by the medical practitioner in treatment of neuralgia of the head and face. They are not local anesthetics to the same extent as cocaine, but are narcotics like morphine. We may use such remedies in preference to cocaine to prevent central excitement. If the pain is from an inflamed pulp, and we cannot allay it by treating the pulp, we should administer a drug having the desired systemic action. We may give morphine, for instance, until local applications have had time to produce the desired local effects. We can use chloral or morphine, for instance, while we use local applications to overcome acute pulpitis and in this way prevent a destructive inflammation. We may also relieve local congestion and pain by the use of a cathartic which will give a revulsive effect, which acts by depleting the pulp and drawing away the circulation locally and similarly affecting the brain centers. A cathartic will cause excessive watery evacuations; a diaphoretic will cause the patient to sweat; thus we extract the water from the blood and relieve the congestion in the brain and the inflamed local area at the same time. By relieving the congestion in the tooth and in the brain at the same time and by a single remedy, we produce a single purpose by a double action. This is probably the most satisfactory anodyne treatment for cases where prompt and complete effects are desired. Simply to apply a little oil of cloves or cocaine to a highly-inflamed pulp reduces the pain locally, perhaps, but leaves the brain centers irritated and excited, and reflex sensations from other parts of the body will cause recurrent pain and keep up the sensation in the brain, and may show its effects in other organs and tissues of the body, even to general systemic prostration.

Systemically, we may cure toothache by mental diversion. The pain in the one brain center may be overcome by producing greater excitement in an other part of the brain. By talking to people in an appropriate and pleasing way we may divert their minds from the subject of greatest interest to them at the time, so that they may not experience much pain from an otherwise painful condition. We may produce similar effects by physical or electrical shocks. By repeated applications of the electric current we may affect other parts of the brain and divert the pain long enough to permit recuperation or resolution, or it may be even dissolution in the diseased parts. We may use the cautery to cure toothache from an inflamed pulp or peridental membrane by producing a blister on some contiguous tissue. The blister diverts the pain to a tissue which is capable of greater injury with less pain and a depleting or revulsive action also is had. If we can produce on the cheek a more powerful irritation than that in the mouth, we can divert the sensation of pain to that part and give the diseased organ in the mouth a chance for resolution. We may also relieve painful conditions by application which tends to produce physical change. By applying moist heat we can relieve tension and relax tissues and so prevent pressure on the nerve endings. Cold is sometimes used for the same purpose, not to relax tissues but to condense them, and keep them from becoming distended.

If we cannot overcome the toothache by any of these anodyne remedies we are compelled to resort to destructive surgical operations. We may extract the tooth; or, if an alveolar abscess we may lance, drain and sterilize it. These are to be classed as pain-relieving measures and within certain limits as anodyne treatments.

One of the most excruciating dental pains ensues from acute pericementitis, and its cause can usually be traced to inflammatory infection from a putrid pulp. In such cases no cure and little relief may be expected until the source of infection has been completely sterilized by instrumental or other therapeutic measures. The most common practice is the local application of a pepper plaster or a lotion of tincture of iodine, or it may be in severe cases a blister. Such remedies always act as local remedies, with a nervous reflex action which has much to do with the successful application. This is the influence produced upon the vascular organs of the tissue implicated through the vaso-motor nerve impulses. By a reflex impulse there is greater tension upon the circulation involved, and consequently revived function. This reflex action is the most valuable feature in the use of local counter-irritants, but it is usually the case that purely-local results are expected or looked for in the use of this class of remedies. It is commonly supposed that only a diversion of the blood-supply is secured in the use of counter-irritants, but the recuperative effect produced by the remedy on the perverted vascular functions through the nervous system is really the only true anodyne effect.

Another class of remedies having exactly opposite physiological reactions are the soothing or demulcent anodynes. These remedies have no considerable physiologic reactions, but act in such a manner as to relieve tension or irritation of the local sensitive nerves. In other words, they act mechanically to protect the exposed tissues and to prevent nervous excitement or stimulation. They are, as a rule, such agents as are insoluble, or, if soluble, such as produce slight excitement of function—oils, waxes, glucrites, mucilages, etc., will fairly exemplify this class. They find a much wider use in general medical than in dental practice. One class of these agents has a considerable and important use in dental practice; this includes the solutions of gummy and resinous substances in volatile solvents. These solutions can be applied to sensitive dentine, exposed pulps, etc., and upon evaporation of the volatile solvent there remains in contact with the sensitive tissue a more or less durable antiseptic protecting layer of inoffensive material which protects the sensitive structures from the mechanical irritation of its environment. At the same time it may be used as a medium to hold medicinal substances in direct contact with a diseased tissue for a sufficiently long period to secure curative action. The whole scheme of pulp dressings or cappings and treatment of sensitive dentine, with such remedies as cavity linings or varnishes, chloropercha, carbolized resin, compound tincture of benzoin and similar remedies are well-known medicinal examples. Generally these substances are used wholly as mechanical applications, but many of them possess good, local anodyne qualities and all may be advantageously combined with chemical reagents of known power and value for curative purposes.

To this class may be added also those corrective and prophylactic remedies used in correcting the effects or influence of perverted secretions on the sensitive tissues of the mouth, antacid mouth washes, antiseptic lotions, and all measures used to prevent the progress of decay of the teeth or inflammatory irritation of the soft tissues. In this class the use of filling materials to prevent the progress of caries and to protect exposed dentine as well as the use of insoluble escharotics like silver nitrate are anodyne remedial agents of great value. In this connection it may be of value for us to take into more serious consideration than we are occasioned to do the varying qualities of our filling materials in respect not only to their durability and convenience of manipulation, but more particularly as to their usefulness in avoiding pain due to loss of the enamel or dentine, the natural protectors of the sensitive pulp organ.

Is it not time that our profession should give more attention to the relief of not only the unendurable pain of acute inflammation of the tooth organs, but to the less pronounced but very continuous irritable conditions incident to thoughtlessness or carelessness in introducing improper filling materials into too close contact with sensitive dentine or pulp organs? Such conditions may produce, through the normal sensible reflexes, systemic conditions of a most serious character, and ought not to be overlooked in our efforts to produce more permanent and durable fillings. Because we are able by the use of efficient local anesthetics to make these operations painlessly, we should not lose sight of the possibility that conditions may result which will bring much suffering to our patients.

